# How Human Is Human Connectional Neuroanatomy?

**DOI:** 10.3389/fnana.2020.00018

**Published:** 2020-04-15

**Authors:** R. Jarrett Rushmore, Sylvain Bouix, Marek Kubicki, Yogesh Rathi, Edward H. Yeterian, Nikos Makris

**Affiliations:** ^1^Department of Anatomy and Neurobiology, Boston University School of Medicine, Boston, MA, United States; ^2^Psychiatric Neuroimaging Laboratory, Harvard Medical School, Brigham and Women’s Hospital, Boston, MA, United States; ^3^Center for Morphometric Analysis, Department of Psychiatry and Neurology, A. Martinos Center for Biomedical Imaging, Massachusetts General Hospital, Harvard Medical School, Boston, MA, United States; ^4^Department of Psychology, Colby College, Waterville, ME, United States

**Keywords:** neuroanatomy, homology, human, monkey, cat, rat, axons, DTI

## Abstract

The structure of the human brain has been studied extensively. Despite all the knowledge accrued, direct information about connections, from origin to termination, in the human brain is extremely limited. Yet there is a widespread misperception that human connectional neuroanatomy is well-established and validated. In this article, we consider what is known directly about human structural and connectional neuroanatomy. Information on neuroanatomical connections in the human brain is derived largely from studies in non-human experimental models in which the entire connectional pathway, including origins, course, and terminations, is directly visualized. Techniques to examine structural connectivity in the human brain are progressing rapidly; nevertheless, our present understanding of such connectivity is limited largely to data derived from homological comparisons, particularly with non-human primates. We take the position that an in-depth and more precise understanding of human connectional neuroanatomy will be obtained by a systematic application of this homological approach.

## Introduction

Understanding the structure of the human brain remains one of the most challenging issues in human neuroscience. While the fundamental architecture of most other organ systems has been determined and the structural organization of their underlying tissues established in great detail, this is not the case in the human brain. The fundamental structure of the human central nervous system, in particular structural brain connectivity, is still not completely delineated.

Different parts of the human central nervous system have been delineated and described using gross anatomical and histological approaches, and according to regional differences in cellular or fibrous composition (e.g., Dejerine and Dejerine-Klumpke, 1895; Vogt and Vogt, 1919; Bailey and von Bonin, 1951; Cajal, 1995; Brodmann, 2006; von Economo et al., 2008). Since the 19th century, the optimal method for dividing the central nervous system into discrete regions has been an object of study and debate. The regional organization of the human brain has been extensively described using post-mortem material and is generally agreed upon. This line of investigation continues using non-invasive brain imaging techniques such as magnetic resonance imaging (MRI), which provides both post-mortem and *in vivo* data (e.g., Toga and Mazziotta, 2002; Walters et al., 2003; Eickhoff et al., 2005).

**Figure 1:**
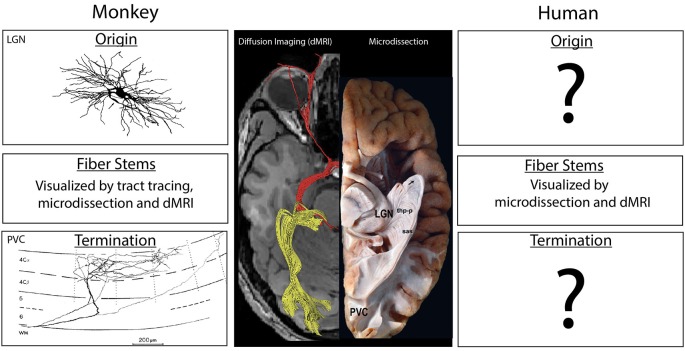
Center: A ventral view of the human brain showing results from diffusion imaging (left side) and microdissection approaches (right side) to show the course of the optic radiations between the lateral geniculate nucleus of the thalamus (LGN) and the PVC. Experimental studies in the macaque monkey (left column) have illustrated the location and morphology of the neurons of origin in the LGN (top) as well as the pattern of termination from individual axons (bottom). The trajectory of the fiber pathway in monkeys can be accurately illustrated in gross anatomical microdissections, *via* diffusion-based MRI (dMRI) tractographic methods and in pathway tracing studies. In the human (right), the trajectory of the pathway may be anatomically indicated by microdissection studies, but these studies often display false positives, as do results from dMRI methods (see the displayed fibers near the eye). The stems of fiber tracts, but not the precise origins and termination of the component axons, can also be illustrated using dMRI methods. Termination patterns and origins of fiber pathways are not directly known in the human, and are inferred from experimental data in the monkey. Diffusion image modified from Hofer et al. (2010). Image of the LGN neuron from Wilson (1989) is copyright 1989, Society for Neuroscience and terminal axon in primary visual cortex (PVC) from Blasdel and Lund (1983) is copyright 1983, Society for Neuroscience. Microdissection image modified with permission from Goga and Türe (2015).

One essential aspect of the human central nervous system structure is virtually unknown: precisely where a specific connection originates and terminates in the brain. Surprising as it may seem, this realization arises from an evaluation of our knowledge of human neuroanatomy. Even among the most studied neuroanatomical systems, e.g., the connections between the lateral geniculate nucleus and the primary visual cortex (PVC), what *direct* evidence do we have in the human? Axonal fibers such as the optic radiations can be observed through microdissection of white matter, e.g., the Klingler technique (Ludwig and Kingler, 1956; Rubino et al., 2005), or inferred from a pattern of degeneration in fibers and cells after a stroke or other localized damage (e.g., Dejerine and Dejerine-Klumpke, 1901). The course of the pathways can also be inferred by tracking the flow of water molecules within axonal bundles using diffusion-based MRI (dMRI) imaging tractography (e.g., Conturo et al., 1999; Mori et al., 1999; Basser et al., 2000; Poupon et al., 2000; Lori et al., 2002). However, none of these methods can directly visualize cellular origins or terminal fields of axons (Figure 1). Both microdissection and dMRI-based methods have difficulties in evaluating branch points or determining trajectories as groups of axons approach each other (Maier-Hein et al., 2017). More problematic is that these methods are unable to determine the trajectory of axons when they enter gray matter and whether they pass through a structure on their way to a deeper layer or region, stop at a proximal point, or branch and turn upon entering a structure (Makris et al., 2002; Schmahmann and Pandya, 2006; Zemmoura et al., 2014; Maier-Hein et al., 2017).

## How Human Is Human Connectional Neuroanatomy?

**Figure 2:**
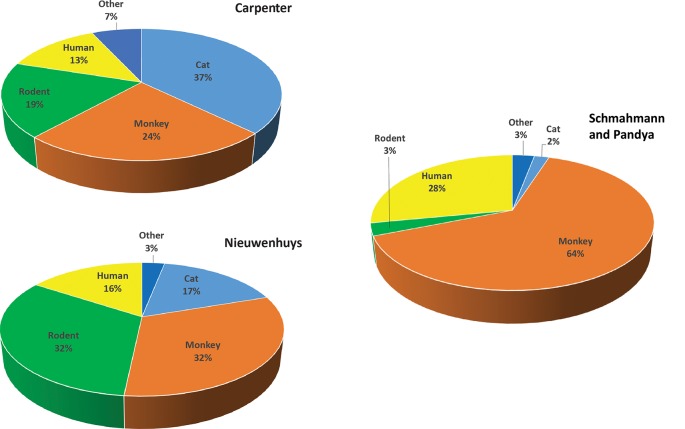
Primary literature in classic human neuroanatomy textbooks according to species. Upper left: percentages derived from primary literature citations found in Carpenter and Sutin (1983). Lower left: percentages derived from Nieuwenhuys et al. (2007). Right: percentages from Schmahmann and Pandya (2006), *Fibers Pathways of the Brain*. For categorization, the term monkey includes all non-human primate species and the term rodent includes rat, mouse, and guinea pig. The term “other” includes species infrequently used, such as rabbit, dog, chicken, ferret, and hamster.

Given this situation, from where does our information on human neuroanatomy, in particular structural brain connectivity, emerge? To address this question, we examined two classic human neuroanatomy texts: Carpenter and Sutin (1983) and Nieuwenhuys et al. (2007). We sorted the primary literature in the references as a metric to determine the sources of information on human brain organization. We found that overwhelmingly, especially regarding structural connectivity, knowledge of human neuroanatomy is obtained through homology, i.e., from experimental animals (Figure 2).

In the Carpenter text, of the primary literature listed in the references section, 37% (781) of studies were performed in cats. Studies in monkeys (mostly macaque species) accounted for 24% (509), while rodents comprised 19% (363). Only 13% (275) of the investigations listed used human material, with the vast majority describing aspects of the human nervous system readily accessible to study such as vascular supply, peripheral nerves, or architectonics. Many of the human investigations described neurological symptoms or neurosurgical approaches and interventions in clinical cases. Several examined post-mortem brain histology in neurological cases to infer the trajectories and termination patterns of degenerated fibers after damage such as that caused by stroke (e.g., Dejerine and Dejerine-Klumpke, 1901). Others delineated fiber systems with fixation or other staining methods (e.g., Dejerine and Dejerine-Klumpke, 1895, 1901) or with microdissection (e.g., Ludwig and Kingler, 1956).

In the more recent Nieuwenhuys text, the proportions of non-human vs. human studies were slightly different. Studies in cats accounted for 17% (576), whereas studies in monkeys and rodents each represented 32% (monkey = 1,098, rodent = 1,122) of the primary literature. Human studies accounted for 16% (543) of the total primary sources. As before, the vast majority of human studies were based on post-mortem analyses, and to this were added non-invasive imaging studies (e.g., positron emission tomography, MRI-based studies, magnetoencephalography) and non-invasive brain stimulation studies (e.g., transcranial magnetic stimulation). A similar analysis of Schmahmann and Pandya (2006) found that 29% of the studies were from human, 65% from the monkey, and the remainder from rat (2.4%), cat (2.2%) and other species (~1.7%).

The human studies cited in these texts do not show precise origins or terminations of fiber pathways. Collectively, these studies provide little direct evidence of structural brain connectivity in humans, except the following approaches. In one, a tracer is applied to slices of the human brain collected quickly after death and kept alive as long as possible (Dai et al., 1998a,b,c). In another, crystals of lipophilic tracers (e.g., DiI; e.g., Galuske et al., 2000; Lai et al., 2018) are placed in regions of the post-mortem human brain and fluorescently visualized after a long incubation period (~12 months in Galuske et al., 2000; 10–14 months in Lai et al., 2018). These approaches, which represent the only direct evidence of connections in the human brain from origin to termination, are limited in that they reveal relatively short connections (5 mm–1.5 cm) within circumscribed regions. Another approach uses the properties of white matter within histological sections to infer connectivity. One such method, 3D polarized light imaging, takes advantage of the fact that myelin is optically anisotropic, and polarized light propagates through it in different ways depending on the orientation of the myelin sheath (Axer H. et al., 2011; Axer et al., 2011a,b; Dohmen et al., 2015). Passing multiple light beams, each with a different polarization allows for bundles of myelinated fibers to be inferred throughout the section. More recent advances in this method measure the degree of attenuation of the polarized light (diattenuation) to infer the orientation of axonal bundles (Menzel et al., 2017, 2019). A related method images brain sections with optical coherence tomography during sectioning, and reconstructs fiber bundles from a series of images after the brain has been sectioned (e.g., Wang et al., 2011, 2014). A final approach with great promise is expansion microscopy, a technique that physically enlarges brain tissue to achieve significant increases in resolution (e.g., Chen et al., 2015; Zhao et al., 2017). These techniques are promising, but not yet comparable in detail and precision to the gold standard of invasive tracing methods in experimental animals.

Thus, the bulk of knowledge that forms the basis of human connectional neuroanatomy is drawn from a variety of non-human species. This fact is not surprising; the homological approach has been and remains a cornerstone of neuroanatomy, including that of the human brain (e.g., Crick and Jones, 1993; Schmahmann and Pandya, 2006). What is surprising is the fact that although not always explicitly acknowledged, homological relationships are the primary source of our knowledge of connectivity in the human brain.

Although gross anatomical and histological features have been well studied in the human brain, precise knowledge of structural connections between specific areas, from origins to terminations, is quite limited. This has been the case since the classic neuroanatomical studies of the 19th century (e.g., Reil, 1809; Broca, 1862; Meynert, 1868; Dejerine and Dejerine-Klumpke, 1895; Burdach, 1922). Because early human neuroanatomists were often physicians (e.g., Brodmann, Dejerine, and others), the focus on human connectional neuroanatomy originated more from a clinical than a strictly neuroanatomical perspective. This perspective meant that neuroanatomical relationships were studied primarily to better interpret how patterns of symptoms could arise from damage to discrete brain structures. To that end, post-mortem human material was used to chart the extent of focal damage induced by pathologies such as stroke, as well as the patterns of degeneration in white and gray matter that occurred in widespread regions of the central nervous system. Connectional relationships were assumed when the primary lesion-induced a pattern of degeneration of white matter, and in some cases gray matter, that appeared to reflect structural connectivity of the primary lesion site with other brain areas (e.g., Dejerine and Dejerine-Klumpke, 1895; Dejerine and Dejerine-Klumpke, 1901).

In parallel, experimental neuroanatomists since the 19th century have focused on brain connections in non-human species with the primary aim of describing fundamental aspects of brain organization rather than clinical relevance. This research has used invasive methods that provide a high level of microscopic detail and employ systematic methodologies aimed at charting connections. Like the clinico-anatomical approach in humans, early studies in animals entailed making lesions in specific parts of the neuraxis, with connectivity inferred based on patterns of degeneration (e.g., Glees, 1946; Nauta and Gygax, 1954; Fink and Heimer, 1967; Nauta and Ebbesson, 1970; Gallyas et al., 1980). This experimental approach, however, was found to include non-specific effects such as false positives due to damage to axons of passage or false negatives due to the lack of a degenerated myelin sheath, which often resulted in imprecise or inaccurate findings (e.g., Schmahmann and Pandya, 2006; Decramer et al., 2018).

In the 1970s degeneration techniques were largely superseded as novel pathway tracing techniques were developed to obviate the limitations of earlier methods. These new techniques involved the intracerebral injection of tracers taken up and transported by neurons (e.g., Cowan et al., 1972; Mesulam, 1976; Mesulam and Rosene, 1977; Rosene and Mesulam, 1978; Mesulam and Rosene, 1979). After an appropriate time for axonal transport of the tracer, the brain was prepared to demonstrate the presence of tracer so that connected areas could be identified, and the strength of connectivity, from origin to termination, qualitatively and quantitatively evaluated. These newer approaches continue to the present, and enormous amounts of information on structural connectivity among different brain regions in different species have been generated. Because the techniques in experimental animals were more precise and controlled than those in humans, and because they provided information on origins and terminations, the more detailed information on brain connections in non-human species was incorporated into the human canon as a foundation of human connectional neuroanatomy. This incorporation of experimental neuroanatomical findings from non-human species into human neuroanatomy has not always been explicit and has led to the inaccurate assumption that a great deal of human connectional neuroanatomy is known directly.

Indeed, human connectional neuroanatomy is based partly on human clinico-anatomical and fiber microdissection studies (and a few studies of very short connections), but largely on homologies generated from anatomical studies in non-human species. The former provides information principally on pathway stems, while the latter comprises the vast majority of information on origins and terminations of brain pathways (Figure 1). Thus, there is scant direct evidence regarding human connectional neuroanatomy, and the data that exist are largely incomplete. Yet there is a widespread misperception that human connectional neuroanatomy is well-established and validated.

## The Importance of Validation and Ground Truth

Awareness of the strong reliance of human neuroanatomy on findings from non-human species is important in the context of current human neuroimaging. The recent advent of non-invasive imaging techniques and the adoption of computer science-based analytical tools has led to a resurgence of human neuroanatomical study and has contributed to the emergence of new fields such as computational neuroanatomy. Non-invasive approaches to neuroanatomy in the human brain have been adopted that use diffusion-based techniques to infer the presence and orientation of fiber bundles *in vivo* (e.g., Conturo et al., 1999; Mori et al., 1999; Basser et al., 2000; Poupon et al., 2000; Lori et al., 2002). These modern imaging approaches to neuroanatomy have revealed with great clarity certain aspects of human fiber system structure. Axons and their myelin sheaths constitute fibers, and fibers in the human brain typically coalesce from their cellular origins into regions in which the main portions, namely the stems of fiber tracts, are found (e.g., Makris et al., 1997, 2002). In these regions where fibers travel together along with the same orientation, their ability to be measured with non-invasive imaging methods such as dMRI is more reliable (e.g., Conturo et al., 1999; Gao et al., 2013; Seehaus et al., 2013). Fibers traveling along the same axis similarly affect water diffusion, reflecting fiber orientation. Diffusion-based tractography methods may then be used to visualize fibers. In this technique, one or more preferred axes of water diffusion for each voxel are measured. A pathway is inferred when adjacent voxels have similar orientations, reflecting the transit of an axonal bundle from one voxel to another (e.g., Conturo et al., 1999; Mori et al., 1999; Basser et al., 2000; Poupon et al., 2000; Lori et al., 2002). Importantly, microdissection techniques and histological analyses have largely validated these data for the largest fiber bundles (e.g., Ludwig and Kingler, 1956; Miklossy et al., 1991).

Although human fiber stems have been validated and are well established using diffusion-based techniques, it is much more difficult for these techniques to define the trajectory of fibers in a voxel when these fibers have a broader range of orientations (e.g., Dauguet et al., 2007; Dyrby et al., 2007; Roebroeck et al., 2008; Thomas et al., 2014; Reveley et al., 2015). As fibers approach gray matter or disperse to travel to different targets, the cohesion in their orientation decreases, along with the ability of diffusion-based techniques to resolve the orientations of the myriad axons contained within the voxel. The reliability of the inferred virtual fiber bundles is accordingly reduced. The ability to resolve fibers near the cortex is also reduced by partial volume effects, where gray and white matter structures share the same voxel (e.g., Caspers and Axer, 2019). Importantly, there are no current techniques in humans to validate the virtual fiber bundles away from regions of high cohesion that define pathway stems.

Connectional *ground truth* comprises the complete structure of fiber bundles, including origins and terminations as well as stems (Figure 1). For the human brain, ground truth for origins and terminations is extremely limited, and, in most instances, does not exist. Consequently, the validity of results from dMRI-based tract-tracing algorithms cannot be compared against ground truth and confirmed. Moreover, the fiber tract structure as inferred from diffusion-based imaging techniques against ground truth cannot be used as feedback to guide the development of better algorithms. The absence of ground truth is thus a major impediment to defining the complete pattern of structural connections in the human brain from origin to termination and represents a barrier that cannot be directly overcome using available technology.

What are the implications of the absence of ground truth for human structural brain connectivity? One important implication is that depending on the acquisition parameters and tractographic analysis tools, the anatomical results provided by diffusion-based tractography can be markedly different for the same structure in the same subject (Makris et al., 2013; Setsompop et al., 2013). A logical consequence is that in the absence of ground truth for human structural connectivity, we cannot with certainty determine whether a tract defined by diffusion-based methods represents a false positive or a true pathway. Similarly, we are unable to evaluate whether the absence of a pathway from a particular structure represents a true negative or a false negative in the human brain (e.g., Gao et al., 2013; Azadbakht et al., 2015; van den Heuvel et al., 2015; Maier-Hein et al., 2017; Sinke et al., 2018).

Ground truth exists for the non-human primate, several species of which have been used in conjunction with invasive tract-tracing methods to comprehensively chart pathways at high resolution (e.g., Schmahmann and Pandya, 2006). The macaque monkey has been one of the dominant models for these neuroanatomical tract-tracing studies. Thus, one way to address the absence of ground truth in human brain connectivity would be to employ a refined homological approach based on non-human primate neuroanatomy. The logic of such an approach is as follows: (1) establish a reliable relationship between experimental neuroanatomical and dMRI connectional data in individual macaque monkeys using empirically developed and validated acquisition and tractographic analysis parameters; (2) formulate a parcellation framework to apply to the macaque monkey brain with a direct correspondence to an established parcellation framework of the human brain for translational purposes; and (3) apply the empirically developed and appropriately scaled dMRI acquisition and tractographic analysis parameters to homological areas of the human brain to examine structural connectivity. It should be noted that such a novel approach would not only provide data for the creation of species-specific atlases (e.g., Mori et al., 2005; Feng et al., 2017; Van Essen and Glasser, 2018), but also would generate data for the creation of homologically-based atlases. Moreover, in addition to providing more precise information on structural connectivity in human and macaque brains, homological atlases would be useful for generating templates to demarcate, localize, and interrelate brain regions and their connections across species.

One approach to compare connections across species that has particular promise is the creation of a connectional fingerprint (e.g., Mars et al., 2018). In this work, the authors use known correspondence in the white matter bundles across species (e.g., human and non-human primates) and diffusion MRI based connectivity profiles of these white matter fiber bundles with gray matter areas to determine homologies between human and primate gray matter areas. Therefore, prior knowledge of white matter correspondence is the key to the determination of gray matter homologies in this work. Nevertheless, the incorporation of tract-tracing data derived from experimental animals into these models could provide a stronger and more direct link between connectional ground truth data and diffusion-based tractographic data that could then be extrapolated to humans.

Other studies relating experimental neuroanatomical data in non-human primates to diffusion-based data have been carried out (van den Heuvel et al., 2015; Donahue et al., 2016; Shen et al., 2019; Van Essen et al., 2019; Hori et al., 2020). These studies have generated measures of connectional strength based on collations of single experiments in which retrograde tracers were injected into a single region and the number of labeled neurons counted. Comparisons were made with dMRI-derived tracts or measures of functional connectivity. These studies provide methodologies important to extending ground truth in the macaque to human connectional neuroanatomy. At the same time, more needs to be done in terms of directly evaluating the white matter pathways (e.g., Schmahmann and Pandya, 2006) to complement data from retrograde tracers, which do not reveal information about where the pathways travel in the white matter, the number of axons involved, or the extent of the terminal arbors.

More studies are needed to better establish homological comparisons between a non-human primate and human connectional neuroanatomy. These comparisons are essential to translate ground truth connectional knowledge based on tract-tracing experiments in non-human primates to the human brain. However, data from extant neuroanatomical tract-tracing in multiple non-human primate species are underutilized and need to be leveraged and used more fully, taking into account the limitations of the methods (e.g., variability in tracer injections, tracer uptake by fibers of passage; Jbabdi et al., 2015). New and existing analytical techniques for diffusion imaging and homological comparisons need to be refined, critically evaluated and tested. Most importantly, the misperception that human connectional neuroanatomy has been solved using classic techniques such as dissection, as well as newer neuroimaging methodologies, needs to be explicitly addressed.

## Conclusion

Although differences in brain structure, connectivity, and function have been documented between non-human primates and human (e.g., Jbabdi et al., 2015), the proposed comparative approach will provide a foundation for the validation of diffusion-based tractography and a more precise approximation of ground truth structural connectivity in the human brain (e.g., Makris et al., 2005; Rilling et al., 2008; Mesulam, 2009; Jbabdi et al., 2013, 2015). Given the current methodological limitations in directly revealing human brain structural connectivity, such a refined homological approach using MRI and dMRI tractographic methods should reduce false positive and negative findings and provide a solid foundation to test hypotheses about the human brain circuit diagram (BCD). Since our knowledge of human connectional neuroanatomy depends so heavily on data from non-human animals, an explicitly comparative approach using dMRI tractography in conjunction with homologically-based atlases will provide a systematic framework for understanding human BCDs with current imaging methods. This approach should make our understanding of human connectional neuroanatomy more accurate, if not necessarily more human.

## Data Availability Statement

The datasets generated for this study are available on request to the corresponding author.

## Author Contributions

RR, NM, and EY wrote the first draft of the manuscript. All authors contributed to manuscript revision, read and approved the submitted version.

## Conflict of Interest

The authors declare that the research was conducted in the absence of any commercial or financial relationships that could be construed as a potential conflict of interest.
